# Supplementary material for: Ventromedial medulla inhibitory neuron inactivation induces REM sleep without atonia and REM sleep behavior disorder
Source: Nat Commun. 2018 Feb 5;9:504. doi: 10.1038/s41467-017-02761-0 (PMC5799338; doi:10.1038/s41467-017-02761-0)
Supplement: Supplementary file 1 — Supplementary Information [file 41467_2017_2761_MOESM1_ESM.pdf]

SUPPLEMENTARY FIGURES

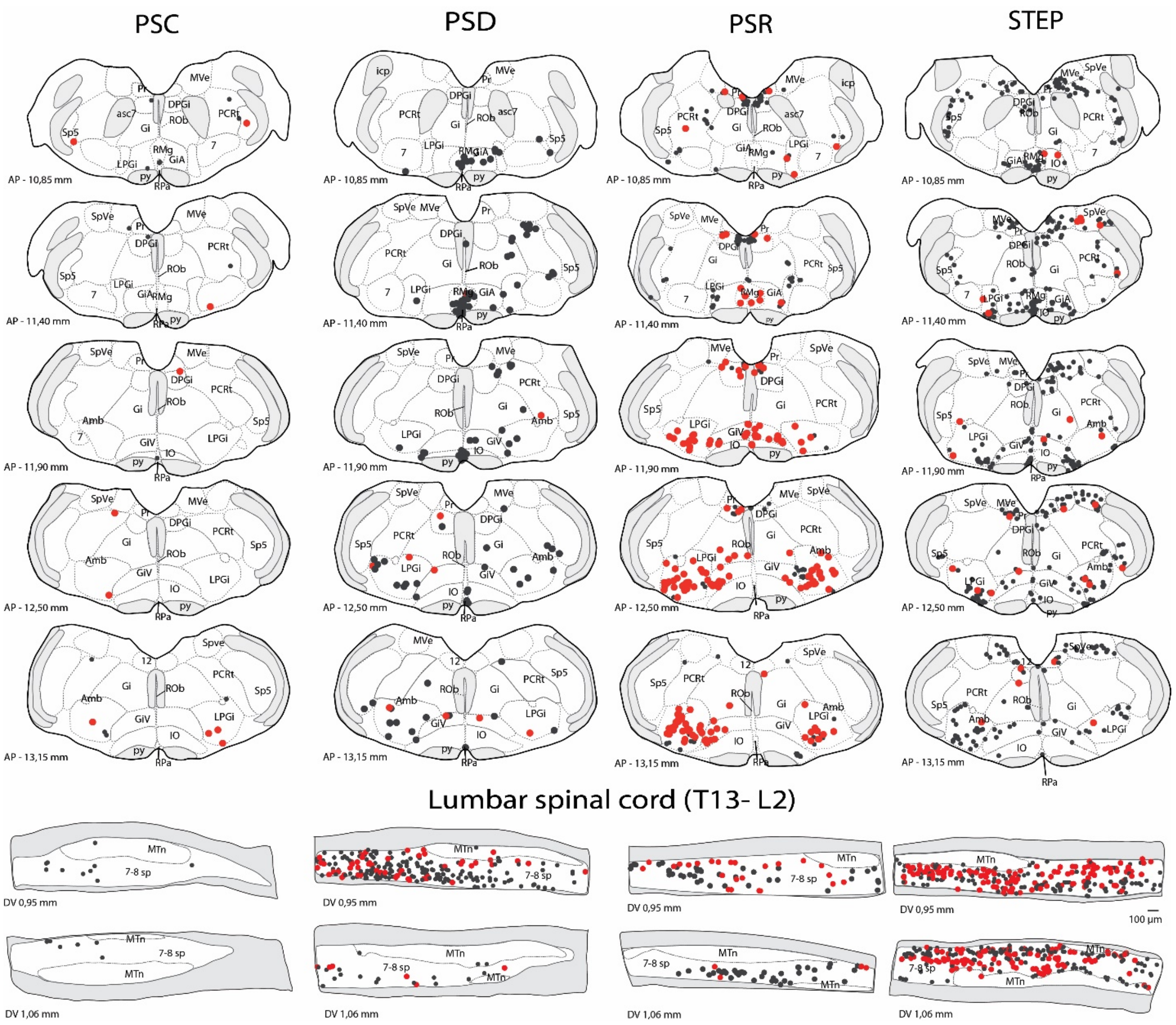

Supplementary Figure 1. Glycine neurons in the vmM, not in lumbar cord, strongly expressed c-Fos during PS rebound.

Drawings rostro-caudally arranged of frontal and horizontal sections of medullary and lumbar spinal neurons respectively comparing the distribution of neurons that expressed c-Fos alone (c-Fos+, black dots) or both c-Fos and GlyT2 mRNA (c-Fos+/GlyT2+; red dots) in representative control (PSC), PS deprived (PSD, second column), PS rebound (PSR, third column) or forced locomotion (STEP, last column) rats. Notice that GlyT2+ vmM neurons are recruited quite exclusively during PSR whereas the activation of lumbar spinal glycine neurons is linked to waking and/or locomotor activity (STEP and PSD). Stereotaxic AP (from Bregma) and DV (from spinal cord surface) coordinates according to Paxinos and Watson atlas are reported for each medullary and spinal sections, respectively.

SUPPLEMENTARY TABLES

	Fos/GlyT2 animals					Fos/FG animals		
	PSC n=4	PSD n=5		PSR n=6		Base line	Rebound	
**PS duration**	18,4 ± 2,7	0,6 ± 0,4	*	59,4 ± 4,1	*#	16,0 ± 2,7	51,8 ± 2	*
**PS percentage**	12,2 ± 1,8	0,4 ± 0,3	*	38,8 ± 2,4	*#	10,7 ± 1,8	34,5 ± 1,3	*
**PS number**	15,5 ± 5,0	2,2 ± 1,7	*	23,8 ± 2,8	#	13,8 ± 2,1	26,3 ± 3,4	*
**PS mean duration**	1,5 ± 0,3	0,1 ± 0,1	*	2,7 ± 0,4	*#	1,2 ± 0,0	2,1 ± 0,3	*
**SWS duration**	85,4 ± 10,2	45,1 ± 4,3	*	65,3 ± 4,0	#	81,0 ± 5,6	64,3 ± 8,3	
**SWS percentage**	56,9 ± 6,8	30,1 ± 2,9	*	42,7 ± 2,5	#	54,0 ± 3,7	42,9 ± 5,5	
**SWS number**	59,8 ± 5,9	78,2 ± 15		39,3 ± 4,0		64,0 ± 4,1	43,5 ± 6,3	
**SWS mean duration**	1,5 ± 0,3	0,8 ± 0,3		1,7 ± 0,1		1,3 ± 0,1	1,6 ± 0,3	
**W duration**	46,4 ± 12,3	104,3 ± 4,7	*	28,3 ± 4,8	#	53,1 ± 6,8	33,9 ± 9,5	
**W percentage**	30,9 ± 8,2	69,5 ± 3,1	*	18,6 ± 3,2	#	35,4 ± 4,5	22,6 ± 6,3	

Supplementary Table 1. Quantitative analysis of the sleep-wake cycle in the rats used for c-Fos/GlyT2 and c-Fos/FG double-labeling experiments. Comparison of the sleep-wake cycle during the last 150min before sacrifice in PSC (n=4), PSD (n=5) and PSR rats (n=7 for c-Fos/GlyT2 experiments; n=4 for c-Fos/FG experiments). Typical parameters calculated for quantifying W, SWS and PS (mean duration in min, mean time percentage, mean episode number and duration in min) were compared to respective baseline values at the same circadian period of the day before PS deprivation. Mean values are displayed with standard errors. Note the reproducibility of the PS deprivation method between the two experiments. For c-Fos/GlyT2 experiments: non-parametric unpaired Mann-Whitney U-tests, * $p < 0,05$ compared to PSC, # $p < 0,05$ compared to PSD. For c-Fos/FG experiments: Wilcoxon Paired tests, *$p < 0.05$ compared to baseline.

	Ctrl-shRNA	vGAT-shRNA	
**PS duration**	118,7 ± 4,6	98 ± 5,9	*
**PS percentage**	8,2 ± 0,3	6,8 ± 0,4	
**PS number**	76,2 ± 4,3	76,4 ± 4,3	
**PS mean duration**	1,6 ± 0,1	1,3 ± 0	*
**SWS duration**	495,6 ± 14,3	509,7 ± 15,7	
**SWS percentage**	34,4 ± 1	35,4 ± 1,1	
**SWS number**	413,0 ± 11,2	443 ± 16,7	
**SWS mean duration**	1,2 ± 0	1,1 ± 0	
**W duration**	825,5 ± 17,5	832,2 ± 17,5	
**W percentage**	57,3 ± 1,2	57,8 ± 1,2	

Supplementary Table 2. Quantitative modification of the sleep-waking cycle induced by the genetic inactivation of GABA/glycine vmM neurons. Comparison of the sleep-wake cycle in Ctrl-shRNA (n=5) and vGAT-shRNA (n=7) rats, 30 days after injections of AAV. For both experimental groups, standard parameters for W, SWS and PS were calculated over 24-h as the mean duration, time percentage, episode number and duration. Durations are expressed in minutes. Mean values are displayed with standard errors. Non-parametric unpaired Mann-Whitney U-test, *p < 0.05 compared to Ctrl-shRNA.
