# Supplementary material for: Ventromedial medulla inhibitory neuron inactivation induces REM sleep without atonia and REM sleep behavior disorder
Source: Nat Commun. 2018 Feb 5;9:504. doi: 10.1038/s41467-017-02761-0 (PMC5799338; doi:10.1038/s41467-017-02761-0)
Supplement: Supplementary file 3 — Description of Additional Supplementary Information [file 41467_2017_2761_MOESM3_ESM.pdf]

Description of Additional Supplementary Files

File Name: Supplementary Movie 1
Description: Channels 1 and 2 (raw nuchal EMG and parietal EEG signals, respectively) and video acquisition are time-locked from the right part of the scrawling window. Notice on the EMG recording that muscle atonia is sustained along the PS episode (marked by the concomitant EEG activation), occasionally overlaid by rare, brief muscle twitches recorded by nuchal electrodes in close body territories as facial or forelimbs muscles (visible on the synchronized video 1).

File Name: Supplementary Movie 2
Description: Three time-locked polysomnographic and video recordings in two representative AAV-shvGAT rats. Channels 1 and 2 (raw nuchal EMG and parietal EEG signals, respectively) and video acquisition are time-locked from the right part of the scrawling window. Almost every PS episode in both rats was marked by an intermittent extinction of muscle atonia, and heterogeneously interrupted by violent movements (Video 2, vigorous movements).

File Name: Supplementary Movie 3
Description: Three time-locked polysomnographic and video recordings in two representative AAV-shvGAT rats. Channels 1 and 2 (raw nuchal EMG and parietal EEG signals, respectively) and video acquisition are time-locked from the right part of the scrawling window. Almost every PS episode in both rats was marked by an intermittent extinction of muscle atonia, and heterogeneously interrupted by violent movements (Video 2, vigorous movements). Occasionally these strong motor behaviors led to the rat's awakening (Videos 3 and 4).

File Name: Supplementary Movie 4
Description: Three time-locked polysomnographic and video recordings in two representative AAV-shvGAT rats. Channels 1 and 2 (raw nuchal EMG and parietal EEG signals, respectively) and video acquisition are time-locked from the right part of the scrawling window. Almost every PS episode in both rats was marked by an intermittent extinction of muscle atonia, and heterogeneously interrupted by violent movements (Video 2, vigorous movements). Occasionally these strong motor behaviors led to the rat's awakening (Videos 3 and 4).
